# Chronobiology-Driven Anti-Aging Strategies for Enhancing Dentists’ Occupational Health and Quality of Life: A Narrative Review

**DOI:** 10.3390/healthcare14060795

**Published:** 2026-03-20

**Authors:** Theodora Kalogerakou

**Affiliations:** Department of Dentistry, School of Health Sciences, National and Kapodistrian University of Athens, 11527 Athens, Greece; tkalogerakou@hotmail.com

**Keywords:** dentists, biological aging, chronomedicine, occupational stress, burnout, musculoskeletal disorders, quality of life, circadian rhythm, lifestyle factors, occupational health

## Abstract

**Background:** Dentists constitute one of the most heavily burdened groups of healthcare professionals, experiencing high levels of musculoskeletal disorders, occupational stress, burnout, and diminished quality of life. Although extensive literature addresses these issues, no study has directly examined biological age or epigenetic markers of aging in this population. This narrative review, informed by systematic methodological principles, seeks to fill this gap by connecting established occupational stressors with contemporary concepts of biological aging and chronomedicine, ultimately proposing a preventive well-being framework specifically for dentists. **Methods:** A narrative review informed by systematic methodology was conducted following PRISMA 2020 guidelines. Searches in PubMed, Scopus, and the Cochrane Library (2015–2025) used combined keywords and MeSH terms related to lifestyle factors, occupational stress, musculoskeletal disorders, quality of life, and wellness among dentists. Of the 943 records identified, 15 met the inclusion criteria and were assessed for outcomes, methodological quality, and relevant risk factors. **Results:** The included studies consistently indicated a significant occupational burden, with musculoskeletal pain, emotional exhaustion, anxiety, and depersonalization as frequent findings. Quality of life was generally moderate to low, especially regarding mental health. Lifestyle patterns were characterized by inadequate sleep, limited physical activity, irregular eating habits, and insufficient recovery. These conditions-chronic stress, poor sleep, inactivity, and suboptimal nutrition-are recognized accelerators of biological aging, implying that the professional demands of dentistry may adversely influence the biological clock. Although none of the studies measured biological age directly, the collective evidence underscores the need for preventive strategies informed by chronomedicine. **Conclusions:** This review highlights a critical gap in the dental literature: the complete absence of biological-age assessment in a professional population exposed to multiple aging accelerators. Integrating occupational health data with modern concepts of biological aging and chronomedicine, the study proposes a targeted preventive framework to regulate biological rhythms, reduce cumulative biological deterioration, and improve the long-term quality of life and professional sustainability of dentists.

## 1. Introduction

Dentists constitute one of the most heavily burdened healthcare professions, routinely exposed to demanding working conditions, psychosomatic strain, and chronic stress that deteriorate performance and well-being [1]. Prolonged static postures, repetitive movements, precision tasks, and continuous clinical and interpersonal pressures contribute to fatigue, musculoskeletal disorders, burnout, and reduced quality of life [2,3,4]. In parallel, biological aging, driven by cumulative cellular damage, epigenetic alterations, chronic low-grade inflammation, oxidative stress, and mitochondrial decline [5,6], is strongly influenced by long-term stress and unhealthy lifestyle patterns. Although biomarkers such as DNA methylation age or epigenetic age acceleration have not yet been applied to dentists, the profession’s inherent demands suggest heightened susceptibility to accelerated aging [6].

Occupational stress is a major driver of biological deterioration. Chronic HPA-axis activation elevates cortisol and disrupts immune, hormonal, and restorative functions, while psychological overload has been associated with telomere shortening and faster cellular aging [7,8]. High levels of burnout, anxiety, and exhaustion—exacerbated during COVID-19—are well documented among dentists [2,9,10]. Musculoskeletal disorders affecting the neck, back, and wrists further contribute to systemic inflammation and reduced job satisfaction [11,12].

Lifestyle patterns common in the profession, including insufficient sleep, low physical activity, irregular eating habits, and inadequate recovery, exacerbate inflammation, impair physiological repair, and negatively affect biological age [13,14,15,16]. Pandemic-related pressures intensified these risks by increasing workload, uncertainty, and psychological strain [2,10]. Evidence demonstrates the protective health impact of Mediterranean diet adherence, regular exercise, and adequate sleep—each associated with reduced inflammation, slower epigenetic aging, and enhanced cellular regeneration [15,16,17]. However, research directly connecting anti-aging strategies to dentists’ biological age remains scarce [13,14,18].

Despite numerous studies documenting musculoskeletal disorders, burnout, and reduced quality of life among dentists, a significant conceptual and methodological gap persists. Existing research primarily describes the symptoms and psychosocial consequences of occupational burden, without examining the cumulative biological impact of these stressors [18,19,20]. In particular, no studies have directly assessed the biological or epigenetic age of dentists, nor have they incorporated chronomedicine as an interpretative and preventive framework in dental occupational health [19,20].

The aim of this narrative review is to synthesize existing evidence on occupational burden, lifestyle factors, and quality of life among dentists and to interpret these findings through the lens of biological aging and chronomedicine. By integrating current knowledge on stress, sleep disturbances, physical inactivity, dietary patterns, and musculoskeletal strain, this review seeks to highlight potential mechanisms linking dental practice to accelerated biological aging and to propose a preventive framework aimed at improving long-term health, resilience, and professional sustainability among dentists.

The originality of the present study lies in its integrative perspective, which connects occupational health research in dentistry with contemporary concepts of biological aging and chronomedicine. While previous studies have primarily focused on isolated outcomes such as musculoskeletal disorders, burnout, or quality of life, this review is the first to synthesize these findings within a unified framework of biological aging mechanisms. By interpreting dentists’ occupational burden through the lens of chronomedicine and lifestyle-related aging pathways, the study introduces a novel conceptual approach that highlights potential links between dental practice and accelerated biological aging, while proposing a preventive framework aimed at improving long-term health and professional sustainability in dentists.

## 2. Materials and Methods

### 2.1. Study Design

This study was designed as a narrative review informed by systematic methodological principles. Although structured search strategies, predefined inclusion and exclusion criteria, and transparent study selection procedures were applied, the present study was not conducted as a fully systematic review because the research question spans multiple domains—occupational health, lifestyle factors, psychological stress, and biological aging—investigated using heterogeneous study designs and outcome measures. Therefore, a narrative review informed by systematic methodological principles was considered the most appropriate approach to synthesize the available evidence.

Concepts such as anti-aging and chronomedicine encompass complex interactions that have not yet been examined using a unified methodological framework in this specific professional population. For this reason, a narrative review structured according to systematic principles was considered the most appropriate methodological approach for synthesizing heterogeneous data, highlighting research gaps, and developing a chronomedical interpretative framework for future research.

### 2.2. Review Objectives

The objective of this review was to explore how lifestyle-related factors and occupational conditions may influence dentists’ biological aging and long-term well-being. In particular, the review aimed to synthesize existing evidence on lifestyle behaviors, including sleep patterns, physical activity, nutrition, and stress management, in relation to dentists’ mental, physical, and occupational health.

In addition, the review examined documented levels of occupational stress, burnout, musculoskeletal disorders, and quality of life among dentists, and analyzed how these occupational burdens may relate to biological mechanisms associated with aging, including chronic low-grade inflammation, oxidative stress, epigenetic age acceleration, and impaired physiological recovery. Finally, the review aimed to identify research gaps and propose future directions for biological-age assessment and preventive strategies in dentistry.

### 2.3. Search Strategy

The review was conducted in alignment with relevant PRISMA 2020 guidelines (Preferred Reporting Items for Systematic Reviews and Meta-Analyses) in order to enhance transparency in reporting the search strategy, study selection, and data synthesis procedures. A comprehensive literature search was performed in three electronic databases: PubMed/MEDLINE, Scopus, and the Cochrane Library (Appendix A).

The search covered studies published between 2015 and 2025 and used combinations of free-text keywords and MeSH terms related to dentists, musculoskeletal disorders, occupational stress, burnout, quality of life, sleep, physical activity, diet, well-being, lifestyle, and mental health. The search strategy was designed to identify primary research examining occupational health and lifestyle-related factors among dental professionals.

Eligible study designs included cross-sectional studies, cohort studies, epidemiological studies, and interventional studies focusing specifically on dentists. Review articles, commentaries, theoretical papers, and studies involving non-clinical populations were excluded in order to ensure that the analysis relied exclusively on original empirical data.

### 2.4. Inclusion and Exclusion Criteria

Studies were considered eligible for inclusion if they involved licensed dental professionals and examined at least one domain related to occupational health or lifestyle patterns. These domains included musculoskeletal disorders, occupational stress, burnout, sleep or fatigue, physical activity, dietary habits, occupational quality of life, or general lifestyle behaviors affecting dentists’ well-being.

Only studies published in English between 2015 and 2025 with accessible full texts were included. Studies involving dental students, dental patients, or other healthcare professionals were excluded in order to ensure that the analysis focused exclusively on active dental practitioners. Articles that lacked methodological transparency, provided limited methodological documentation, or did not report primary empirical data were also excluded.

### 2.5. Study Selection Process

The study selection process followed a three-stage screening procedure designed to ensure methodological transparency and consistency. Initially, duplicate records identified across databases were removed. Subsequently, titles and abstracts were screened independently in order to identify potentially relevant studies. In the final stage, full-text articles were assessed to confirm eligibility based on the predefined inclusion and exclusion criteria.

This multi-stage screening procedure ensured that only studies directly relevant to the research objectives and methodological standards of the review were included in the final analysis.

### 2.6. Data Extraction and Quality Assessment

Data extraction focused on key characteristics of the included studies in order to allow systematic comparison and synthesis of the evidence. The extracted information included the country of origin, study design, sample size and characteristics, assessment tools used, prevalence of musculoskeletal disorders and burnout, quality-of-life outcomes, and reported associations between lifestyle factors and occupational health indicators.

The methodological quality of the included studies was evaluated using the Joanna Briggs Institute (JBI) Critical Appraisal Tool. This standardized assessment instrument evaluates aspects such as population clarity, sampling procedures, measurement validity, control of confounding factors, and statistical adequacy. The use of this tool allowed the methodological robustness and potential risk of bias of each study to be systematically evaluated.

The methodological rigor and reliability of the included studies were evaluated using standardized appraisal criteria, with the full assessment presented in Table 1. Overall, most studies demonstrated moderate methodological quality, with clearly defined populations, appropriate measurement instruments, and adequate statistical analyses. However, several studies were limited by cross-sectional designs, reliance on self-reported data, and potential selection bias associated with convenience sampling. Despite these limitations, the overall methodological consistency of the included studies supports the reliability of the identified patterns regarding occupational burden, lifestyle factors, and quality-of-life outcomes among dentists.

### 2.7. Sensitivity Analysis

Sensitivity analyses were conducted in order to evaluate the robustness and stability of the synthesized findings. These analyses examined whether the overall patterns observed in the data remained stable when specific categories of studies were excluded or when thematic variables were grouped differently.

In particular, sensitivity analyses explored how the removal of specific thematic categories of studies, the grouping of lifestyle variables into broader domains, and the exclusion of studies from geographically overrepresented regions influenced the overall interpretation of the findings. This approach allowed the identification of potential evidence gaps and strengthened the internal validity of the review.

### 2.8. PRISMA Flow and Study Selection

**Figure 1:**
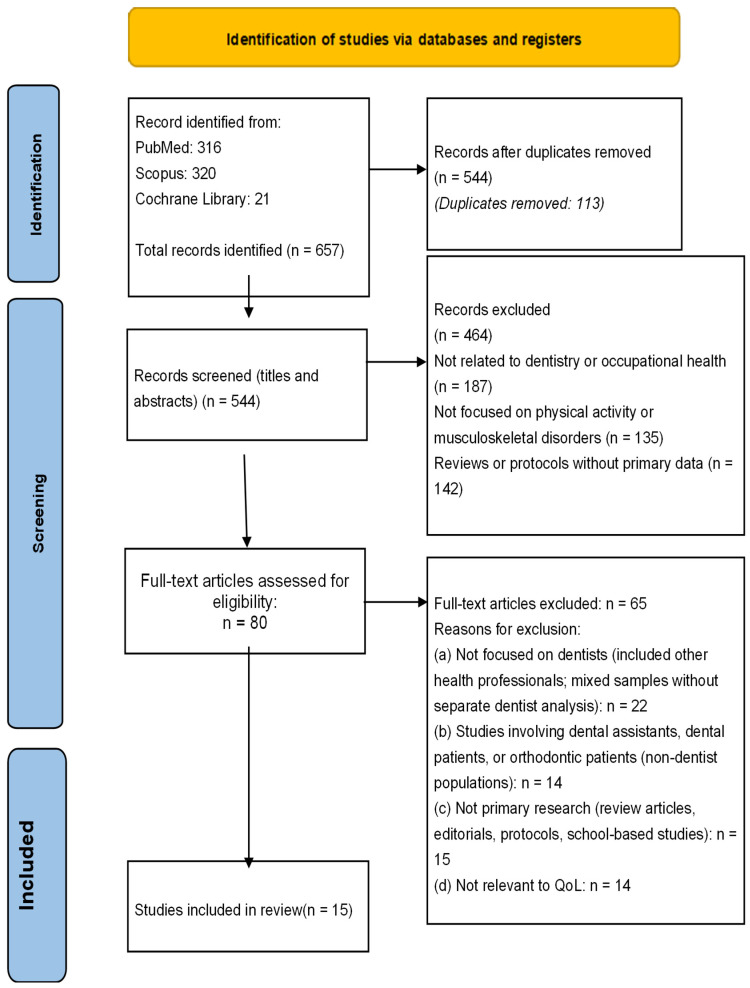
Flowchart for the results of the search strategy.

The flowchart illustrates the study selection process followed in this systematic review, according to the stages of identification, screening, eligibility assessment, and final inclusion. Initially, a total of 657 records were identified through database searches, including PubMed (316 records), Scopus (320 records), and the Cochrane Library (21 records). After the removal of 113 duplicate records, 544 studies remained for screening based on titles and abstracts. During this stage, 464 records were excluded, primarily because they were not related to dentistry or occupational health (n = 187), were not focused on physical activity or musculoskeletal disorders (n = 135), or consisted of reviews or protocols without primary data (n = 142). Subsequently, 80 full-text articles were assessed for eligibility. Of these, 65 articles were excluded due to several reasons, including lack of focus on dentists as the main population, inclusion of mixed healthcare professional samples without separate analysis for dentists, inclusion of non-dentist populations, or lack of relevance to quality of life outcomes. Ultimately, 15 studies met all inclusion criteria and were included in the final systematic review. This flowchart clearly presents the transparency and methodological rigor of the study selection process, Figure 1.

## 3. Results

In total, 15 studies from different geographical regions were included in the review, examining various dimensions of dental professionals’ occupational health. The main characteristics of the studies are summarized in Table 2.

The methodological rigor and reliability of the included studies were evaluated using standardized appraisal criteria, with the full assessment provided in Table 1.

The included studies covered a broad range of themes, encompassing (a) musculoskeletal disorders, (b) occupational burnout and work-related stress, (c) quality of life, and (d) lifestyle factors influencing occupational health, Table 3.

The sensitivity analysis indicates that, although the magnitude of effects varies across analytical scenarios, the overall direction of the findings remains stable. Excluding specific categories of studies attenuates certain estimates but does not alter the conclusion that dentists face substantial physical and psychological workload-related risks. The influence of ergonomic and lifestyle-related factors emerges as particularly robust, strengthening associations with musculoskeletal disorders and mental strain. Overall, the results demonstrate strong methodological robustness and external validity of the review findings.

### 3.1. Musculoskeletal Disorders

Musculoskeletal disorders (MSDs) emerged as the most frequent and stable occupational health problem among dentists, regardless of geographical region or type of work environment. The overall studies demonstrate a particularly high incidence of pain in the neck, back, and upper extremities, suggesting that musculoskeletal burden is a structural feature of dental practice [12,21,22,27,32,33].

The severity and frequency of symptoms showed a clear exposure-response relationship. Increased daily clinical workload, high patient volumes, prolonged static postures, and limited physical activity were repeatedly associated with more severe symptoms and functional limitations [21,22,27,32]. In contrast, regular exercise, work breaks, and ergonomic training were protective, although their implementation in daily practice was often insufficient [12]. Overall, the findings suggest that MSDs act as a chronic and cumulative occupational stressor. Although no study directly assessed biomarkers of biological aging, the pattern of chronic pain, reduced function, and work-related fatigue is stable with mechanisms of accelerated physiological wear and tear associated with prolonged musculoskeletal strain.

### 3.2. Burnout and Psychological Stress

Burnout and psychological stress were the second dominant theme, with converging evidence of high levels of emotional exhaustion, depersonalization, and perceived stress in dentists [2,23,24,25,29].

The intensity of burnout was mainly influenced by rehabilitation factors. Insufficient sleep duration and quality, limited physical activity, and lack of rest time were stably associated with increased levels of psychological burden [2,23,25]. In contrast, healthy lifestyle behaviors appeared to partially mitigate the risk. Demographic and occupational factors, such as gender and years of experience, influenced vulnerability, but did not overshadow the role of workload and lifestyle [2].

The organizational environment proved to be decisive. Higher levels of burnout were recorded in structures with increased demands, limited autonomy, and low job satisfaction [24]. In particular, the study by Meyerson et al. [30] showed that neurobiological sensitivity can intensify burnout, but can also be transformed into a source of job satisfaction in supportive environments. Overall, the complex of chronic stress, sleep disturbance, and emotional fatigue aligns with mechanisms of cumulative physiological load (allostatic load).

### 3.3. Quality of Life

Quality of life (QoL) was investigated directly in four studies and indirectly in several others through functional and psychosocial indicators. Overall, dentists reported lower quality-of-life scores, especially in the domains of mental health, social functioning, and environment [13,28,31,34].

Working conditions and workload emerged as the main determinants of QoL variation, while specialty alone did not appear to play a decisive role [31]. Social support factors, such as marital status, served as protective factors in some populations [13]. The observed differences in quality of life reflect the cumulative effect of chronic occupational stress and not individual work exposures.

### 3.4. Lifestyle Factors

Lifestyle factors were a cross-cutting theme that affected both physical and mental health. Insufficient physical activity, reduced sleep, unhealthy eating habits, and alcohol or tobacco consumption were stably associated with increased burnout, psychological distress, and reduced quality of life [2,13,14,25].

Conversely, adopting healthy behaviors appeared to enhance occupational resilience. Lifestyle factors did not operate in isolation, but interacted with occupational demands, enhancing or moderating the overall risk burden. The findings highlight modifiable behavioral factors as critical intervention targets for dentists’ long-term occupational well-being.

**Figure 2:**
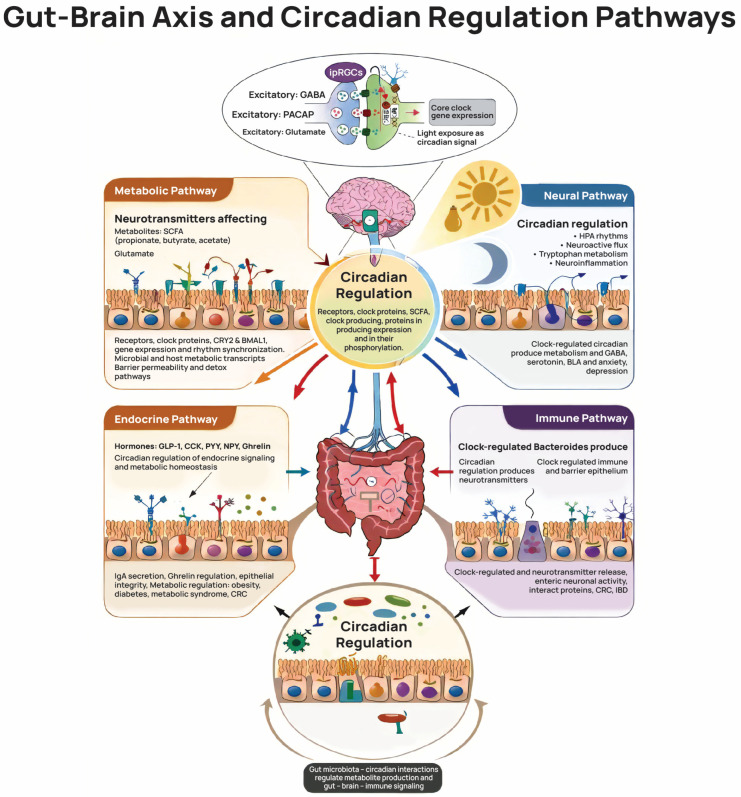
Circadian Regulation and the Brain–Gut–Microbiome Axis.

Figure 2 summarizes the conceptual relationships between occupational burden, lifestyle factors, and mechanisms of biological aging in dentists, as identified in the reviewed studies.

## 4. Discussion

Rather than depicting isolated occupational or lifestyle-related problems, the findings synthesized in this review reveal a coherent and interrelated pattern of physical, psychological, and behavioral burdens that characterize contemporary dental practice. Across diverse geographic regions and healthcare systems, dentists stably exhibit high musculoskeletal strain, elevated levels of occupational stress and burnout, disrupted sleep and recovery patterns, and compromised quality of life. Importantly, these factors do not operate independently but interact dynamically, reinforcing one another and generating cumulative physiological load over time. When interpreted through the lens of biological aging and chronomedicine, this constellation of occupational stressors aligns with well-established mechanisms of accelerated biological deterioration, including chronic low-grade inflammation, neuroendocrine dysregulation, impaired circadian regulation, and reduced regenerative capacity. Thus, the present findings support a shift from a descriptive understanding of dentists’ occupational health challenges toward an integrative, mechanistic framework that conceptualizes dentistry as a profession with heightened vulnerability to premature functional aging.

This review demonstrates that dentists are exposed to a complex, multifactorial, and cumulative occupational burden, which includes high levels of musculoskeletal strain, occupational stress, emotional exhaustion, psychological overload, and systematically reduced quality of life. The findings of the fifteen included studies do not describe isolated or fragmented problems, but reflect a coherent pattern of interacting factors that simultaneously affect the physical, mental, and functional health of dentists. The above findings are in line with data from the Greek dental population, which has documented a high incidence of occupational burnout, with a decisive role for factors such as increased workload, uncertainty, and disruption of professional balance, both before and during the COVID-19 pandemic [3].

These mechanisms are discussed to contextualize the findings biologically. Of particular note is the fact that, despite extensive documentation of these burdens, no studies have been identified that directly assess markers of biological age, epigenetic deterioration, or molecular aging in the dental population. This gap is critical, given that the dominant risk factors that emerge-sleep disturbances, chronic stress, limited physical activity, inadequate nutrition, and inadequate rehabilitation-have been internationally recognized as powerful accelerators of biological aging [6,35,36,37]. This discussion interprets the findings in light of modern biological, epigenetic, and chronomedical theories, proposing a unified framework for anti-aging interventions tailored to the specificities of the dental profession [38].

It should be emphasized that the present review does not provide direct evidence of accelerated biological aging in dentists, as none of the included studies assessed biological age markers such as epigenetic clocks, telomere length, inflammatory biomarkers, or mitochondrial function. Rather, the findings highlight occupational and lifestyle factors that have been associated with biological aging mechanisms in the broader biomedical literature. Therefore, the proposed relationship between dentistry-related occupational burden and biological aging should be interpreted as a theoretically plausible hypothesis requiring direct empirical investigation.

### 4.1. Sleep, Circadian Rhythm, and Biological Aging

Sleep is a fundamental regulator of homeostasis, immune balance, and physiological aging processes. Chronic sleep deprivation has been associated in the broader biomedical literature with telomere shortening, increased oxidative stress, impaired metabolic regulation, and dysregulation of the hypothalamic–pituitary–adrenal axis [15,39]. In addition, circadian dysregulation and fragmented sleep have been linked to epigenetic aging signatures and metabolic dysfunction in several population studies [40].

Studies by Slabšinskienė et al. [2], Badrasawi et al. [23], and Yuh et al. [25] indicate that dentists often report shorter sleep duration than recommended, difficulty disengaging from work-related stress, and fragmented or non-restorative sleep. These patterns have been associated with higher emotional exhaustion, elevated perceived stress, reduced professional resilience, and increased musculoskeletal burden [41]. While these findings do not directly demonstrate biological aging processes in dentists, they highlight occupational and lifestyle factors that have been linked to biological aging pathways in the broader literature. Given that sleep supports essential processes such as immune regulation, inflammation control, and mitochondrial maintenance [42], persistent disruption may represent a potential contributor to long-term biological dysregulation.

Beyond sleep duration, circadian rhythm integrity influences neuroendocrine, metabolic, and immunological regulation, including interactions mediated through the brain–gut axis [43]. The gut microbiome itself exhibits circadian oscillations that are influenced by sleep–wake cycles, timing of food intake, and light exposure. Disruption of this “chronobiome” has been associated with metabolic and neuroendocrine imbalance in several studies [44], suggesting that circadian instability may represent a potential vulnerability factor in high-demand professions such as dentistry. In this context, chronomedically informed strategies—including the reduction of prolonged clinical schedules, limitation of late-night digital exposure, and the establishment of stable sleep routines—may represent biologically relevant preventive approaches [45,46,47].

Sleep and circadian rhythms operate as components of an integrated regulatory system rather than independent physiological processes [15,39]. Through coordinated neuroendocrine signaling, metabolic regulation, immune surveillance, and microbial interactions, this system acts as a temporal organizer of physiological activity, aligning cellular repair, energy metabolism, hormonal secretion, and immune responses with environmental and behavioral cues [40].

Central to this regulatory network is the bidirectional communication between the central nervous system, the gastrointestinal tract, and the gut microbiome, commonly described as the brain–gut–microbiome axis [43,44]. This axis integrates neural, endocrine, immune, and microbial signaling pathways, allowing circadian information to be transmitted across multiple organ systems. Microbial metabolites may influence neuroendocrine signaling, inflammatory tone, and metabolic regulation, reinforcing the systemic nature of circadian organization [43,44].

Circadian cues—primarily shaped by sleep–wake cycles, light exposure, and meal timing—contribute to the synchronization of hormonal secretion, immune responses, and microbial rhythms [42,45,46]. Proper alignment of these cues supports cellular repair processes, maintains inflammatory balance, and sustains metabolic flexibility. Conversely, chronic disruption of circadian rhythms, irregular work schedules, and persistent occupational stress—conditions frequently reported in demanding healthcare professions such as dentistry—may interfere with this regulatory system [35,36]. Such disruptions have been associated with dysregulated cortisol patterns, altered immune responses, gut microbiome imbalance, and increased oxidative and inflammatory burden [6,35,36,37].

Within this framework, the integrative chronobiological perspective illustrated in Figure 2 provides a conceptual model through which occupational stressors in dental practice can be interpreted. Sleep disruption, circadian misalignment, and occupational strain may interact and potentially contribute to cumulative biological stress over time. However, it should be emphasized that the present review synthesizes indirect evidence from occupational health studies and broader biological research, and does not provide direct measurements of biological aging in dentists. Future longitudinal and biomarker-based studies are needed to clarify the extent to which these mechanisms translate into measurable biological aging processes within this professional population.

### 4.2. Diet, Antioxidant Protection, and Metabolic Load

Although none of the primary studies directly assessed dietary intake in detail, the broader biomedical literature demonstrates strong associations between nutritional patterns, systemic inflammation, and biological aging processes [48]. Diets rich in antioxidants and anti-inflammatory components, such as the Mediterranean diet, have been associated with reduced epigenetic age acceleration and lower levels of chronic inflammation in population-based studies [49]. Conversely, frequent consumption of fast food, irregular eating patterns, meal skipping, and insufficient intake of antioxidant-rich foods have been linked to increased oxidative stress and metabolic dysregulation [50].

The findings of Abraham et al. [13] and Alrayes et al. [14] suggest that dentists may frequently adopt irregular dietary patterns, including meal skipping and suboptimal nutritional choices, largely due to increased workload and time constraints. Similar patterns have been reported among other healthcare professionals, where demanding work schedules may negatively influence dietary habits and overall health behaviors [51]. While these studies do not directly evaluate biological aging markers, they highlight lifestyle factors that have been associated with metabolic and inflammatory pathways relevant to biological aging. Nutritional strategies aimed at supporting antioxidant defenses, improving omega-3 fatty acid intake, and maintaining structured meal patterns have therefore been proposed as potentially beneficial lifestyle interventions [18,52,53,54].

The dietary patterns observed among dentists may reflect adaptive responses to occupational demands rather than deliberate, evidence-based nutritional choices. High clinical workload, time pressure, irregular schedules, and limited recovery periods can contribute to meal skipping, reliance on energy-dense but micronutrient-poor foods, and reduced intake of antioxidant-rich nutrients [18,52]. These patterns should therefore be interpreted within the broader context of occupational constraints that may shape long-term metabolic exposure.

Over time, repeated engagement in irregular eating patterns and diets low in antioxidants, omega-3 fatty acids, and bioactive micronutrients has been associated in the broader literature with increased oxidative stress, impaired metabolic regulation, and mitochondrial dysfunction [53]. Such metabolic disturbances are also linked to chronic low-grade inflammation and may influence epigenetic regulatory mechanisms, including DNA methylation patterns and metabolic signaling pathways. These mechanisms provide a potential biological explanation for how long-term nutritional imbalance may contribute to physiological vulnerability.

Within this context, nutrition can be viewed as an important modifiable factor influencing metabolic balance, immune regulation, and cellular repair processes [48,50,52]. Adequate nutritional intake may support antioxidant defense systems and physiological recovery, whereas persistent nutritional imbalance may increase metabolic burden and reduce the organism’s ability to counteract occupational stressors.

Importantly, metabolic stress associated with suboptimal dietary patterns may interact with other occupational characteristics frequently reported in dentistry, such as reduced physical activity and chronic musculoskeletal strain. These factors have been associated with increased inflammatory signaling and reduced physiological recovery capacity in several studies [52,53]. In combination, such conditions may contribute to a biological environment characterized by sustained inflammatory activation, often described in the literature as “inflammaging” [53].

Taken together, these observations suggest that nutritional behavior may represent an important component of occupational health in dentists. Rather than serving solely as a behavioral variable, dietary patterns may influence metabolic efficiency, inflammatory regulation, and long-term physiological resilience. However, further studies incorporating direct nutritional assessment and biological aging biomarkers are needed to clarify the extent to which these mechanisms are reflected in measurable biological aging processes among dental professionals.

### 4.3. Physical Activity, Musculoskeletal Strain, and “Inflammaging”

Physical activity is widely recognized as an important protective factor against several biological processes associated with aging, exerting beneficial effects on metabolic regulation, inflammatory balance, and cellular repair mechanisms [55,56,57]. Regular exercise has been shown to improve mitochondrial function, enhance cellular energy metabolism, and reduce chronic low-grade inflammation, thereby supporting physiological homeostasis and resilience against age-related functional decline [55,56]. In addition, growing evidence suggests that sustained physical activity may be associated with slower epigenetic aging, reflected in reduced DNA methylation age acceleration and more favorable molecular aging profiles in population-based studies [57,58].

Empirical studies by Alghadir et al. [32], Ohlendorf et al. [26], Alsoleihat et al. [22], and Eddhaoui and Syed [12] indicate that dentists frequently report limited levels of physical activity, prolonged static or flexed working postures, and minimal opportunities for movement during clinical practice. These occupational characteristics are associated with a high prevalence of musculoskeletal pain and functional limitations. Although musculoskeletal disorders are often considered localized biomechanical conditions, emerging evidence suggests that chronic musculoskeletal strain may also be associated with systemic inflammatory responses, a process sometimes described in the literature as “inflammaging,” which has been linked to biological aging pathways [59].

In this context, interventions such as regular movement breaks during clinical work, strengthening of postural and stabilizing muscle groups, and low- to moderate-intensity aerobic exercise may have importance not only from an ergonomic perspective but also in relation to broader physiological regulation. Physical activity has been shown to influence inflammatory signaling, mitochondrial function, and metabolic balance, factors that have been associated with healthy aging trajectories [55,60]. The potential importance of exercise for occupational resilience in dentistry is further supported by a recent systematic review indicating that regular physical activity may reduce occupational stress, improve psychological well-being, and support functional capacity among dentists in the post-pandemic professional environment [61].

The high prevalence of musculoskeletal disorders among dentists may therefore reflect the cumulative effects of biomechanical load, limited physical activity, and demanding work environments. Recurrent pain, neuromuscular fatigue, and reduced mobility may contribute to sustained inflammatory signaling and impaired physiological recovery processes [59,62]. While these findings do not directly demonstrate biological age acceleration in dentists, they highlight occupational conditions that have been associated with inflammatory and metabolic pathways relevant to biological aging. Within this framework, physical activity and ergonomic interventions can be viewed as important strategies that may support both musculoskeletal health and broader physiological resilience. Furthermore, persistent physical strain often coexists with psychological stress in dental practice, potentially amplifying both somatic and emotional dimensions of occupational load and reinforcing cumulative stress responses over time [2,23,25].

### 4.4. Stress Management and Psychological Resilience

Chronic psychological stress has been widely associated in the broader biomedical literature with several biological processes related to aging, including neuroendocrine dysregulation, immune activation, and persistent low-grade inflammation [8,62]. Sustained activation of the hypothalamic–pituitary–adrenal (HPA) axis may lead to altered cortisol rhythms, impaired immune surveillance, and increased oxidative stress, mechanisms that have been linked to physiological dysregulation and age-related disease risk [8,35].

Consistent with these mechanistic insights, occupational studies suggest that dentists frequently experience substantial psychological burden, including anxiety, emotional exhaustion, depersonalization, and depressive symptoms [2,23,24,25]. For example, Slabšinskienė et al. [2] and Badrasawi et al. [23] report associations between chronic occupational stress, insufficient recovery, and burnout dimensions, while Ciğerim et al. [24] and Yuh et al. [25] indicate that demanding work environments and lifestyle constraints may further increase psychological strain among dental professionals.

In the broader literature, chronic psychological stress has also been associated with biological markers that are commonly used to study aging processes, such as telomere shortening and epigenetic age acceleration [7,8,62]. These observations highlight the systemic biological impact of prolonged stress exposure beyond purely psychological effects. However, it should be noted that none of the studies included in the present review directly assessed biological aging biomarkers in dentists.

Persistent emotional exhaustion and depersonalization are often interpreted as indicators of prolonged exposure to occupational stressors that exceed adaptive capacity, potentially leading to cumulative physiological burden conceptualized as increased allostatic load [58,62]. Within this framework, psychological stress may represent an important contributor to long-term physiological strain in high-demand professions.

Several stress-management strategies have been proposed as potentially beneficial in mitigating these processes. Mindfulness-based interventions and controlled breathing techniques have been associated with improvements in stress regulation, modulation of inflammatory signaling, and restoration of autonomic balance in various populations [63,64]. Similarly, interventions emphasizing empathy, emotional awareness, and self-compassion have demonstrated beneficial effects in reducing burnout symptoms and enhancing psychological resilience among healthcare professionals, including dentists [65,66].

At a practical level, brief breathing exercises, structured relaxation practices, and organizational psychosocial support systems may help reduce the physiological burden associated with chronic stress exposure by attenuating neuroendocrine activation and inflammatory signaling [66,67]. Such strategies may be particularly relevant in dentistry, where high workload and time constraints frequently limit opportunities for prolonged recovery [23,25].

Overall, the evidence suggests that chronic psychological stress may represent an important occupational factor influencing neuroendocrine regulation, immune balance, and inflammatory signaling in dental professionals. While these mechanisms have been linked to biological aging processes in the broader scientific literature, direct biomarker-based evidence in dentists remains limited. Future studies incorporating longitudinal designs and biological aging indicators are therefore needed to clarify the extent to which occupational stress in dentistry translates into measurable biological aging trajectories.

Finally, psychological stress in dentistry often coexists with ergonomic constraints and chronic musculoskeletal strain, creating a complex interaction between mental and physical stressors. This interaction may amplify both somatic and emotional dimensions of occupational burden, highlighting the importance of integrated interventions that simultaneously address psychological resilience, physical workload, and organizational conditions in dental practice [26,32,59,60,61].

### 4.5. Ergonomics, Functional Capacity, and Aging

Musculoskeletal disorders in dentists are not exclusively mechanical in origin. Chronic ergonomic strain has been associated with neuromuscular fatigue, reduced physical endurance, and increased inflammatory signaling, conditions that may contribute to physiological changes resembling early functional aging processes [59,68]. Evidence from the studies included in this review indicates that prolonged exposure to unfavorable working postures and repetitive strain is linked to functional limitations and musculoskeletal discomfort among dental professionals [69,70].

In the broader literature, chronic musculoskeletal strain has been associated with reduced muscular strength, diminished endurance, and altered neuromuscular coordination—changes that are commonly observed in age-related functional decline [69,70]. When such conditions persist over extended professional careers, they may influence occupational longevity, limit adaptive capacity, and reduce the ability of dentists to sustain demanding clinical workloads over time.

Ergonomic optimization, particularly when combined with regular movement and exercise-based interventions, has been suggested as an important strategy for maintaining musculoskeletal health and functional capacity in dental professionals [71,72]. Such approaches may reduce biomechanical load while also supporting neuromuscular efficiency and overall functional resilience. In addition, physical activity and ergonomic improvements have been associated with reductions in pro-inflammatory signaling and improved physical performance in several studies [71,72].

Beyond purely physical determinants, psychosocial workplace environments also appear to influence stress responses and functional outcomes. Supportive organizational contexts, adequate psychosocial resources, and balanced workloads have been shown to moderate physiological stress responses and contribute to improved occupational sustainability among healthcare professionals, including dentists [70,72].

Taken together, these observations suggest that musculoskeletal strain, physical workload, and workplace conditions interact in complex ways that may influence long-term functional capacity in dental professionals. However, further longitudinal research incorporating biological and functional aging markers is required to clarify the extent to which these occupational factors translate into measurable aging-related changes.

### 4.6. Psychosocial Factors and Social Support

Psychosocial factors are widely recognized as important determinants of both mental health and overall physiological regulation. In the broader literature, social isolation has been associated with increased inflammatory activity, reduced stress resilience, and biological processes that have been linked to aging-related physiological changes [73,74]. Studies by Abraham et al. [13] and Afshar et al. [28] indicate that dentists with limited social support networks report lower quality of life and higher levels of burnout, a finding that has also been supported by more recent evidence [75]. Social support may therefore play a protective role by moderating psychological stress and attenuating neuroendocrine and inflammatory responses [76,77,78]. The importance of supportive psychosocial environments is also reflected in studies among academic health professionals, which suggest that burnout and reduced well-being are associated with chronic occupational stress and insufficient support systems [4].

Psychosocial support has been described as an important buffer against occupational stress by influencing physiological stress responses, neuroendocrine regulation, and inflammatory activity [73,74]. In dental professionals, reduced social support has been associated with poorer quality of life and increased vulnerability to occupational stress, highlighting the relevance of relational and organizational contexts in shaping psychological and physiological resilience [74,75].

From a broader aging perspective, social connectedness has been proposed as a protective factor that may help mitigate stress-related physiological burden, support emotional well-being, and contribute to long-term functional resilience [73,74]. These psychosocial influences may interact with biological regulatory systems involved in stress adaptation, including immune function, neuroendocrine balance, and allostatic load regulation [74,75]. However, it should be noted that direct evidence linking psychosocial factors to biological aging biomarkers in dentists remains limited, and further research is needed to clarify these potential relationships.

### 4.7. Connection with the Biological Clock

According to López-Otín et al. [6], biological aging is driven by interconnected mechanisms, including genomic instability, epigenetic alterations, chronic inflammation, mitochondrial dysfunction, metabolic dysregulation, and disrupted intercellular communication [58,79]. These processes are influenced by a wide range of environmental, behavioral, and physiological factors that interact over time. Several occupational and lifestyle characteristics frequently reported in dental professionals—such as chronic stress, sleep disturbances, limited physical activity, irregular dietary patterns, and prolonged musculoskeletal strain—have been associated in the broader literature with biological pathways relevant to aging processes [80,81,82,83].

Taken together, the findings synthesized in this review suggest that the professional environment of dentistry may involve multiple occupational stressors and lifestyle constraints that could influence physiological systems linked to biological aging. However, it should be emphasized that the studies included in this review did not directly assess biological aging biomarkers, and therefore the proposed relationship should be interpreted as a conceptual integration of occupational health evidence with established biological aging mechanisms.

Viewed collectively, the occupational exposures and lifestyle patterns identified in this review converge on several regulatory systems involved in maintaining physiological homeostasis, including neuroendocrine signaling, inflammatory balance, mitochondrial function, and epigenetic regulation [80,81]. Dentistry may therefore represent a professional context in which several factors commonly associated with aging-related biological pathways—such as chronic stress, circadian disruption, reduced physical activity, metabolic imbalance, and prolonged musculoskeletal strain—coexist and potentially interact over time [81,82].

This convergence highlights the cumulative nature of occupational exposures in dentistry, where repeated or long-term exposure to multiple stressors may contribute to physiological strain and reduced recovery capacity. From this perspective, chronomedically informed preventive strategies may play an important role in supporting long-term health, functional capacity, and occupational sustainability among dental professionals. Such approaches emphasize the importance of aligning behavioral, occupational, and physiological rhythms in order to support biological resilience and overall well-being across the professional lifespan [80,83].

### 4.8. Final Considerations

In conclusion, the findings of the large-scale epidemiological study by Stamatakis et al. [84] demonstrate a strong concordance with the conclusions of the present review, supporting a unified and biologically coherent interpretation of lifestyle-related health risk [80,83,84]. Both lines of evidence stably indicate that adverse health outcomes and accelerated biological deterioration do not result from isolated behaviors, but from the cumulative and synergistic interaction of disrupted sleep, insufficient physical activity, and suboptimal dietary patterns. Importantly, the study by Stamatakis et al. [84] shows that even moderate, yet concurrent, improvements across these three domains are associated with a substantial reduction in all-cause mortality, highlighting that the health benefit is multiplicative rather than merely additive.

Within the context of this review, these findings acquire particular relevance for dentists, a professional population exposed to sustained occupational stress, circadian disruption, musculoskeletal strain, and irregular lifestyle habits. Our synthesis indicates that sleep quality, habitual physical activity, and dietary structure function as key regulators of biological age, modulating inflammatory load, neuroendocrine balance, metabolic regulation, and functional resilience. The convergence between population-level mortality data and profession-specific evidence strengthens the biological and clinical validity of an integrated, chronomedical preventive framework. Overall, the evidence supports the conclusion that long-term adherence to adequate and stable daily behaviors represents a more effective and biologically meaningful strategy for preserving longevity, functional capacity, and sustainable quality of life than the pursuit of isolated, extreme, or short-term lifestyle interventions [84].

Recent evidence from studies in dental students indicates that musculoskeletal symptoms, sleep disturbances, and sociodemographic factors significantly influence work-related quality of life, suggesting that occupational health risks associated with dentistry may emerge early in professional development [85,86]. Furthermore, the documented association between musculoskeletal disorders and burnout symptoms highlights the multidimensional nature of occupational burden in dentistry and supports the need for early preventive strategies aimed at improving long-term professional sustainability and well-being [87].

### 4.9. Future Directions

The findings underscore the need for research that: (a) directly assesses the epigenetic age of dentists; (b) employs longitudinal designs to track the progression of biological deterioration over time; (c) conducts interventional trials targeting sleep, nutrition, physical activity, and ergonomic practices; (d) explores the impact of relaxation techniques and mindfulness on biological aging; and (e) develops a specialized “Dentist Biological Age Index” (DBAI) to quantify and monitor aging trajectories in this professional population.

### 4.10. Limitations and Risk of Bias

This narrative review possesses several notable strengths. It follows a rigorous PRISMA-based methodology, includes exclusively primary studies conducted on dentists, synthesizes findings across diverse geographic and socioeconomic settings, and explicitly connects the evidence to contemporary scientific knowledge on biological aging. These elements enhance the robustness, relevance, and interdisciplinary value of the review.

However, certain limitations must be acknowledged. None of the included primary studies measured epigenetic or biological aging markers, creating a substantial evidence gap. The heterogeneity of measurement tools across studies limits comparability, while the predominance of cross-sectional designs restricts causal inference. Additionally, the reliance on self-reported data introduces potential recall and reporting biases.

The main risks of bias identified include selection bias from convenience samples, self-reporting, and recall bias in self-reported data; limitations of the cross-sectional study design; inadequate control of confounding factors; and possible publication bias.

An important limitation of the present review is that none of the included studies directly measured biological age or molecular aging markers. Consequently, the discussion of biological aging mechanisms should be interpreted as a conceptual integration of occupational health findings with established biological aging pathways rather than as direct empirical evidence.

## 5. Conclusions

This narrative review shows that dentists are among the most biologically and psychologically burdened health professionals, presenting high rates of musculoskeletal disorders, burnout, mood disturbances, and reduced quality of life. Lifestyle factors such as insufficient sleep, low physical activity, irregular dietary patterns, and limited recovery appear to further aggravate these risks. These conditions correspond to mechanisms associated with accelerated biological aging, including chronic inflammation, oxidative stress, and impaired physiological recovery. However, no studies have yet directly assessed biological or epigenetic aging in dentists, highlighting an important research gap. Clinically, the findings support the implementation of preventive strategies focusing on sleep regulation, physical activity, ergonomic practices, stress management, and healthy nutrition. Such interventions may help reduce cumulative biological burden and improve dentists’ long-term health and professional sustainability.

## Figures and Tables

**Table 1:** Inclusion and exclusion criteria used for study selection.

Criteria	Inclusion Criteria	Exclusion Criteria
Population	Licensed dentists and active dental professionals	Dental students, dental assistants, dental patients, or other healthcare professionals
Study focus	Studies examining occupational health, musculoskeletal disorders, burnout, stress, sleep, physical activity, dietary habits, lifestyle behaviors, or quality of life in dentists	Studies not related to dentists’ occupational health or lifestyle factors
Study design	Primary empirical studies (cross-sectional, cohort, epidemiological, or interventional studies)	Review articles, editorials, commentaries, theoretical papers, protocols
Publication characteristics	Studies published in English between 2015–2025 with accessible full text	Non-English publications or studies without accessible full text
Methodological quality	Studies with clearly described methodology and validated assessment instruments.	Studies with insufficient methodological description or lack of primary data

**Table 2:** Study characteristics and key findings/outcomes of included studies (n = 15).

Author (s), Country, Year	Study Design	Sample & Population	Setting	Exposure	Comparator	Statistical Significance	Limitations	Outcomes & Key Findings
1. Eddhaoui & Syed, Qatar, 2025 [12]	Cross-sectional	n = 330 dentists in primary care	Primary health care	MSDs, ergonomics	None	Significant	Self-reported, cross-sectional	MSD prevalence 78.6%; 45.5% required sick leave. Despite high ergonomic knowledge (>70%), actual practice < 60%. Emphasizes need for routine exercise & applied ergonomic training.
2. Benfaida et al., Morocco, 2024 [21]	Cross-sectional	n = 210 private dentists	Clinical	MSDs, workload, inactivity	Working hours, patients/day	Significant	Convenience sample	Very high MSD prevalence. Strong correlations with working hours, number of patients/day, and physical inactivity. Calls for ergonomic training & physical activity programs.
3. Alsoleihat et al., Jordan, 2024 [22]	Cross-sectional	n = 450 dentists	Mixed	MSDs, overtime, inactivity	Exercise vs. no exercise	Significant	Cross-sectional	High prevalence of pain in upper limbs, neck & lower back. Strong associations with overtime work and lack of exercise. Ergonomic interventions urgently needed.
4. Badrasawi et al., Palestine, 2024 [23]	Cross-sectional	n = 320 dentists	Clinical	Stress, sleep duration	Sleep duration groups	Significant (*p* < 0.05)	Self-reported	81% reported moderate/high stress; 48% high emotional exhaustion. Sleep duration significantly related to burnout (*p* < 0.05). Recommendations: sleep-optimization programs & stress-management training.
5. Ciğerim et al., Turkey, 2024 [24]	Cross-sectional	n = 517 dentists in 3 service types	Clinical	Burnout, workload	Service type	Significant	Cross-sectional	Higher burnout & depression in dentists working in ODHCs. Key factors: limited free time, low income satisfaction, heavy workload. Need for structural reorganization and mental health support.
6. Yuh et al., South Korea, 2024 [25]	Nationwide cross-sectional	n = 1028 dentists	National sample	Lifestyle behaviors	Healthy vs. unhealthy	Significant	Self-reported	Anxiety, depression & burnout linked to low exercise, increased alcohol/smoking, poor sleep. Healthy lifestyle behaviors reduce psychological distress.
7. Ohlendorf et al., Germany, 2020 [26]	Cross-sectional	dentists	Clinical	Ergonomic load, working posture, clinical tasks	None	Significant	Cross-sectional design; inclusion of dental students; self-reported data	High prevalence of musculoskeletal disorders, particularly in the neck and lower back, associated with prolonged static postures and inadequate ergonomic practices. Dentists not applying ergonomic principles experienced higher physical strain. The study highlights the importance of ergonomic training and regular movement breaks as preventive strategies to reduce cumulative occupational load and long-term functional deterioration.
8. Boreak et al., Saudi Arabia, 2023 [27]	Cross-sectional	n = 260 dentists	Clinical (public & private)	Endodontic workload	Loupes vs. no loupes	Significant	Cross-sectional	MSDs in 61.5% of dentists performing endodontics. 80% reported magnification improved manual skills. Loupes reduced psychological stress (49.2%) and physical strain (64.6%).
9. Afshar et al., Brazil, 2022 [28]	Cross-sectional	n = 403 public-sector dentists	Public health clinics	Occupational hazards	High vs. low risk	Significant	Self-reported	High exposure to chemical, ergonomic & psychosocial hazards. Lower QoL observed in those reporting high occupational risk. Exercise & preventive measures linked to better outcomes.
10. Díaz-Caballero & Evaristo-Chiyong, Peru, 2022 [29]	Cross-sectional analytic	n = 168 dentists	Clinical	Burnout	Gender, experience	Significant	Sample size	Burnout prevalence: 28.57%. High emotional exhaustion (90.47%) & depersonalization (98.09%). Lower burnout risk in females (aPR = 0.53, *p* = 0.044) and dentists with 11–20 years’ experience (aPR = 0.30, *p* = 0.017).
11. Slabšinskienė et al., Lithuania, 2021 [2]	Cross-sectional	n = 380 dentists	Mixed	Sleep, activity, recovery	Lifestyle groups	Significant	Cross-sectional	Burnout positively associated with poor sleep, low physical activity & low recovery time. Exercise & relaxation activities were protective factors.
12. Meyerson et al., Israel, 2020 [30]	Cross-sectional	n = 235 dentists	Clinical	Sensory sensitivity	High vs. low SPS	Mixed	Self-reported	High sensory processing sensitivity associated with higher burnout but also greater job satisfaction in supportive environments. Highlights need for individualized stress-management interventions.
13. Abraham et al., UAE, 2018 [13]	Cross-sectional	n = 135 dentists	Clinical/Private practice	Specialty, marital status	Specialists vs. general	Significant (*p* < 0.05)	Small sample	Specialists had significantly higher QoL than general practitioners (*p* < 0.05). Married dentists scored higher in social & environmental domains. Work–life balance essential for improving QoL.
14. Zeinabadi et al., Iran, 2018 [31]	Cross-sectional	n = 288 dentists (public & private sectors)	Mixed	Workload, rest time	Specialists vs. general	Not significant	Cross-sectional	No major QoL differences between general dentists and specialists. Workload and rest time more influential than specialty. Importance of schedule restructuring emphasized.
15. Alghadir et al., Saudi Arabia, 2015 [32]	Cross-sectional	n = 250 dentists (various specialties)	Clinical	MSDs, clinical hours	Gender, specialty	Significant	Self-reported	High prevalence of MSDs (neck, back, upper limbs). Significant associations with gender, age, specialty, clinical hours. Ergonomic training & physical activity proposed as preventive factors.

**Table 3:** Sensitivity Analysis Summary for detailed outcomes.

Sensitivity Condition	Impact on Findings	Effect Description
Excluding MSD-focused studies	Overall estimates of occupational burden decrease	Removal of high-prevalence MSD data (78–100%) lowers the apparent physical strain but does not alter the conclusion that dentists experience significant workload-related health risks.
Excluding burnout-focused studies	Stress-related trends weaken	Without burnout data (emotional exhaustion > 60% in several samples), the psychological burden appears less severe, though still present due to QoL and lifestyle findings.
Grouping studies by lifestyle factors	Associations with mental health become more pronounced	Clustering variables (sleep, physical activity, alcohol, smoking) strengthens the pattern that lifestyle behaviors substantially affect psychological well-being and resilience.
Removing studies from specific regions (e.g., Middle East)	Minimal change in overall direction of results	Despite geographic differences, findings remain internationally stable, indicating strong external validity of stress and MSD patterns.
Focusing exclusively on full WHOQOL-BREF outcomes	Slight reduction in observed burnout intensity	QoL scores often mask underlying emotional exhaustion and depersonalization, suggesting that burnout-specific tools capture the problem more sensitively.
Excluding small-sample studies (e.g., n < 50)	Reduced statistical variability	Larger studies maintain the same direction of effects, but associations (e.g., age, gender) become more stable and less sample-dependent.
Combining ergonomic and workload-related variables	Stronger identification of risk factors	Extended working hours, patient load, and insufficient ergonomic practice stably predict MSDs and mental fatigue.

## Data Availability

No new data were created or analyzed in this study. Data sharing is not applicable to this article.

## Appendix A

**Search Strategies**

The following material provides a detailed and transparent description of the search strategies applied in each database. The search strings were tailored to each database’s structure and indexing system to ensure reproducibility and methodological clarity, in line with PRISMA 2020 recommendations.

**PubMed/MEDLINE**

The literature search in PubMed/MEDLINE was conducted using a combination of Medical Subject Headings (MeSH) and free-text terms (Title/Abstract), as follows:

(dentists[MeSH Terms] OR dentist*[Title/Abstract]) AND (burnout[MeSH Terms] OR burnout[Title/Abstract] OR "quality of life"[MeSH Terms]) AND (work*[Title/Abstract] OR practice[Title/Abstract]) AND

(“2015”[Date - Publication]: “2025”[Date - Publication])

**Scopus**

The Scopus search was performed using the Advanced Search option and the TITLE-ABS-KEY field:

TITLE-ABS-KEY (dentist*) AND TITLE-ABS-KEY (burnout OR "quality of life”) AND TITLE-ABS-KEY (work*OR practice) AND PUBYEAR > 2014 AND PUBYEAR < 2025 AND (LIMIT-TO (LANGUAGE, “English”))

**Cochrane Library**

Due to the specific scope and structure of the Cochrane Library, a more targeted search strategy was applied, focusing on title and abstract fields:

(dentist*):ti AND ("quality of life" OR burnout):ti,ab
